# Image quality in three-dimensional (3D) contrast-enhanced dynamic magnetic resonance imaging of the abdomen using deep learning denoising technique: intraindividual comparison between T1-weighted sequences with compressed sensing and with a modified Fast 3D mode wheel

**DOI:** 10.1007/s11604-024-01687-0

**Published:** 2024-11-06

**Authors:** Masahiro Tanabe, Yosuke Kawano, Atsuo Inoue, Keisuke Miyoshi, Haruki Furutani, Kenichiro Ihara, Mayumi Higashi, Katsuyoshi Ito

**Affiliations:** Department of Radiology, Yamaguchi University Graduate School of Medicine, 1-1-1 Minami-Kogushi, Ube, Yamaguchi 755-8505 Japan

**Keywords:** Compressed sensing, Deep learning denoising, Contrast-enhanced dynamic MR, Fast 3D mode

## Abstract

**Purpose:**

To assess the image quality of a modified Fast three-dimensional (Fast 3D) mode wheel with sequential data filling (mFast 3D wheel) combined with a deep learning denoising technique (Advanced Intelligent Clear-IQ Engine [AiCE]) in contrast-enhanced (CE) 3D dynamic magnetic resonance (MR) imaging of the abdomen during a single breath hold (BH) by intra-individual comparison with compressed sensing (CS) with AiCE.

**Methods:**

Forty-two patients who underwent multiphasic CE dynamic MRI obtained with both mFast 3D wheel using AiCE and CS using AiCE in the same patient were retrospectively included. The conspicuity, artifacts, image quality, signal intensity ratio (SIR), signal-to-noise ratio (SNR), contrast ratio (CR), and contrast enhancement ratio (CER) of the organs were compared between these 2 sequences.

**Results:**

Conspicuity, artifacts, and overall image quality were significantly better in the mFast 3D wheel using AiCE than in the CS with AiCE (all *p* < 0.001). The SNR of the liver in CS with AiCE was significantly better than that in the mFast 3D wheel using AiCE (*p* < 0.01). There were no significant differences in the SIR, CR, and CER between the two sequences.

**Conclusion:**

A mFast 3D wheel using AiCE as a deep learning denoising technique improved the conspicuity of abdominal organs and intrahepatic structures and the overall image quality with sufficient contrast enhancement effects, making it feasible for BH 3D CE dynamic MR imaging of the abdomen.

## Introduction

In multiphasic contrast-enhanced (CE) three-dimensional (3D) dynamic magnetic resonance (MR) imaging of the abdomen during breath-holding, which has become an essential technique for the screening and assessment of abdominal diseases [1, 2], high spatial and contrast resolution imaging is expected. However, these goals are not fully achieved due to the trade-off relationship with acquisition time, which is limited [3, 4]. Several studies have shown methods to decrease the imaging time in breath-hold (BH) abdominal MRI, such as parallel imaging (PI) using sensitivity encoding [5] and compressed sensing (CS) using random k-space undersampling [6]. CS can reduce the imaging time by employing image sparsity and incoherent k-space sampling in the data acquisition process [7], which can be allocated to improve the spatial resolution. In addition, contrast resolution can be improved by combining a deep learning-based denoising algorithm (advanced intelligent clear-IQ engine [AiCE]) [8, 9].

The Fast 3D mode wheel technique is a recently developed method that collects signals in a wheel-like fashion from the center of the k-space segmented into fan-shapes in the phase-encoded-slice-encoded plane [10, 11], thereby reducing the imaging time and making it applicable to dynamic abdominal imaging. More recently, the modified Fast 3D mode wheel technique, in which signals are collected in a wheel shape with sequential data filling in the k-space in the phase-encode-slice-encode plane (modified Fast 3D mode wheel with sequential data filling; mFast 3D wheel), has been available in clinical practice. This technique was developed to improve the image blurring observed in the original Fast 3D mode and can also be combined with AiCE for image denoising, enabling high spatial and contrast resolution imaging. Therefore, the purpose of this study was to conduct an intraindividual comparison of image quality in BH 3D CE dynamic MR imaging of the abdomen using AiCE as a deep learning denoising technique between high spatial and contrast-resolution T1-weighted sequences with CS and with mFast 3D wheel.

## Materials and methods

### Study population

**Fig. 1:**
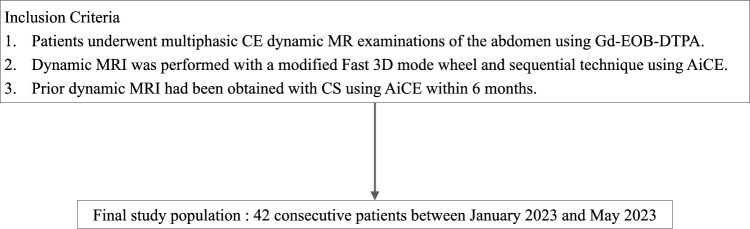
Inclusion criteria for patient selection. *CE* contrast-enhanced, *CS* compressed sensing, *AiCE* advanced intelligent clear-IQ engine

This study received approval from our institutional review board, which waived the requirement for written informed consent for this retrospective data analysis. Our radiology database was searched to identify patients who met the following inclusion criteria: (a) underwent multiphasic CE dynamic MR examinations of the abdomen using Gd-EOB-DTPA (EOB Primovist; Bayer Pharma, Osaka, Japan) between January 2023 and May 2023; (b) received dynamic MRI with the mFast 3D wheel using AiCE; (c) prior dynamic MRI was obtained with CS using AiCE within 6 months for intra-individual comparison. Finally, 42 patients met these criteria and were included in this study (28 men and 14 women; age range, 22–84 years; mean age, 68.5 years) (Fig. 1). The reasons for abdominal MRI were surveillance or post-therapeutic follow-up of hepatocellular carcinoma in patients with chronic hepatitis or cirrhosis (n = 32), screening or suspected liver metastasis in oncology patients (n = 9), and further evaluation of suspected hepatic hemangioma (n = 1). Demographics of study population are shown in Table 1.

**Table 1:** Demographics of study population

Characteristics	Patients, n (%)
Total number of patients	42
Man/woman	28 (67) /14 (33)
Age (years) *	68.5 ± 12.2
Underlying liver disease	
Chronic hepatitis B	6 (14)
Chronic hepatitis C	8 (19)
Chronic hepatitis B + C	1 (2)
Non-B, non-C liver cirrhosis	8 (19)
Alchoholic liver cirrhosis	8 (19)
Autoimmune hepatitis	1 (2)
Healthy liver	10 (24)
Child–Pugh score	
5	31 (74)
6	6 (14)
7	4 (10)
10	1 (2)

^*^Data are means ± standard deviations

### MRI technique

All patients underwent MRI examinations using a 3 T system (Vantage Centurian; Canon Medical Systems, Otawara, Japan) equipped with a 16-channel body phased-array coil. BH 3D CE dynamic T1-weighted gradient echo (GRE) imaging with fat suppression (FS) was conducted before and after the intravenous administration of Gd-EOB-DTPA (0.1 mg/kg). The contrast material was injected using a power injector at an injection rate of 1.0 mL/sec flushed with 20 mL of saline. Multiphasic images were obtained in the arterial phase (AP), portal venous phase (PVP), transitional phase (TP), and hepatobiliary phase (HBP) after intravenous injection. The AP images were acquired using a bolus tracking technique adjusted by monitoring the intensity of the thoracic aorta. The PVP, TP and HBP images were acquired at 60 s, 180 s and 20 min after contrast injection, respectively. The patients underwent both dynamic imaging using CS with AiCE and a mFast 3D wheel using AiCE, but not on the same day. The interval between the two examinations was within 6 months, with the CS sequence first. The MRI parameters for dynamic imaging using CS with AiCE and the mFast 3D wheel using AiCE are shown in Table 2. The spatial resolution was set as high as possible and optimized for each sequence within approximately the same imaging time. Hence, the in-plane resolution was set slightly higher in the mFast 3D wheel using AiCE (0.50 × 0.50 mm) than in CS with AiCE (0.63 × 0.63 mm).

**Table 2:** MR parameters for dynamic imaging using CS with AiCE and a mFast 3D wheel using AiCE

MR parameters	CS	mFast 3D wheel
TR/TE (msec)	3.1/1.2	3.4/1.3
Flip angle (degrees)	12	12
Bandwidth (Hz/pixel)	781	781
FOV (mm^2^)	320 × 380	320 × 380
Slice thickness (mm)	1.5	1.5
Matrix size	512 × 608	640 × 760
Voxel size (mm^3^)	0.63 × 0.63 × 1.5	0.50 × 0.50 × 1.5
CS factors (in-plane × slice)	2.5 × 1.4	N/A
SPEEDER factors (in-plane × slice)	N/A	2.5 × 1.4
Number of excitation	1	1
Acquisition time (sec)	17	18
AiCE level	4	4

*CS* compressed sensing, *mFast 3D wheel* modified fast 3D mode wheel with sequential data filling, *AiCE* advanced intelligent clear-IQ engine, *TR* repetition time, *TE* echo time, *FOV* field of view

## Image analysis

### Qualitative analyses

**Fig. 2:**
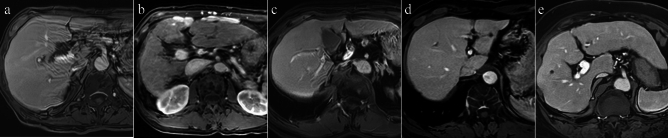
Examples of the overall image quality. **a** score 1; extremely poor and non-diagnostic image quality with extremely severe artifacts, **b** score 2; poor image quality with severe artifacts, **c** score 3; moderate image, **d** score 4; good image quality with mild artifacts, **e** score 5; excellent image quality with no artifacts

Dynamic MR images from both CS with AiCE and the mFast 3D wheel using AiCE were independently reviewed by three radiologists with 3, 12, and 36 years of experience in abdominal MRI who were blinded to the types of images and the date of MR examinations. Visual evaluation scores were rated on a Likert scale ranging from 1 to 5 using AP and PVP images comprehensively. Reading scores of ≥ 3 were considered sufficient for clinical use: image noise and artifacts due to motion and edge enhancement (1 = extremely severe and non-diagnostic, 2 = severe, 3 = moderate, 4 = mild, 5 = no noise), conspicuity of abdominal organs and intrahepatic structures (image sharpness; edge of liver and pancreas and intrahepatic vasculatures), and overall image quality (1 = extremely poor and non-diagnostic, 2 = poor, 3 = moderate, 4 = good, 5 = excellent) (Fig. 2). Regarding conspicuity of intrahepatic structures, the intrahepatic portal veins and hepatic veins were evaluated in the PVP.

The timing of arterial phase was evaluated by another radiologists with 22 years of experience in abdominal MRI using a 3-point scale as follows: 1 = early (unclear enhancement of the hepatic artery, no appreciable enhancement of the portal vein); 2 = optimal (obvious enhancement in the hepatic artery, partial enhancement of the portal vein with minimal enhancement of the hepatic parenchyma and no contrast visualized in the hepatic veins); and 3 = late (obvious enhancement of the hepatic parenchyma and presence of contrast in the hepatic veins) [12].

### Quantitative analyses

A radiologist with 5 years of experience in abdominal MRI, who knew the type of image but did not know the results of the qualitative evaluation, placed a circular or oval region of interest (ROI) on the target organs; the ROI was as large as possible (200 – 400 mm^2^ for the liver, 150 – 250 mm^2^ for the pancreas, 150 – 350 mm^2^ for the erector spinae muscle). In the liver, 3 ROIs were placed in the right lobe (2 ROIs) and left lobe (1 ROI). The ROIs of the liver were placed at the level of umbilical portion of portal vein. In the pancreas, as a representative hypervascular abdominal organ, 2 ROI measurements were performed at the pancreatic parenchyma. The ROIs were taken care to place in the same position for the two images. All ROIs were placed to avoid large vessels and artifacts. Additionally, the ROI was placed on both sides of the erector spinae muscle at the level of the hepatic hilum. These measurements were used to compare the signal intensity ratio (SIR), signal-to-noise ratio (SNR) of the organs, the contrast ratio (CR), contrast enhancement ratio (CER) and contrast-to-noise ratio (CNR) of the organs between dynamic images using CS with AiCE and the mFast 3D wheel using AiCE. Each ratio was calculated according to the following equation: SIR = signal intensity (SI) of the organ/SI of the muscle, SNR = SI of the organ/SD of the organ, CR = (SI of the organ – SI of the muscle)/SI of the organ, CER = (post SI of the organ – pre-SI of the organ)/pre-SI of the organ, and CNR = (SI of the organ – SI of the muscle)/SD of the organ [13–15].

### Statistical analyses

The SPSS software program (SPSS, version 27.0; IBM, Armonk, NY, USA) was used to perform all statistical analyses. Quantitative and qualitative analyses were performed using the Wilcoxon signed-rank test. Fisher’s exact test was used to compare the proportion of timing in the arterial phase between CS with AiCE and the mFast 3D wheel using AiCE. *P* values of < 0.05 were considered to indicate statistical significance. Interobserver agreement for visual subjective scores was evaluated using Fleiss kappa statistics. The kappa value was assessed as follows: 0.81–1.00, excellent agreement; 0.61–0.80, substantial agreement; 0.41–0.60, moderate agreement; 0.21–0.40, fair agreement; < 0.20, poor agreement.

## Results

**Fig. 3:**
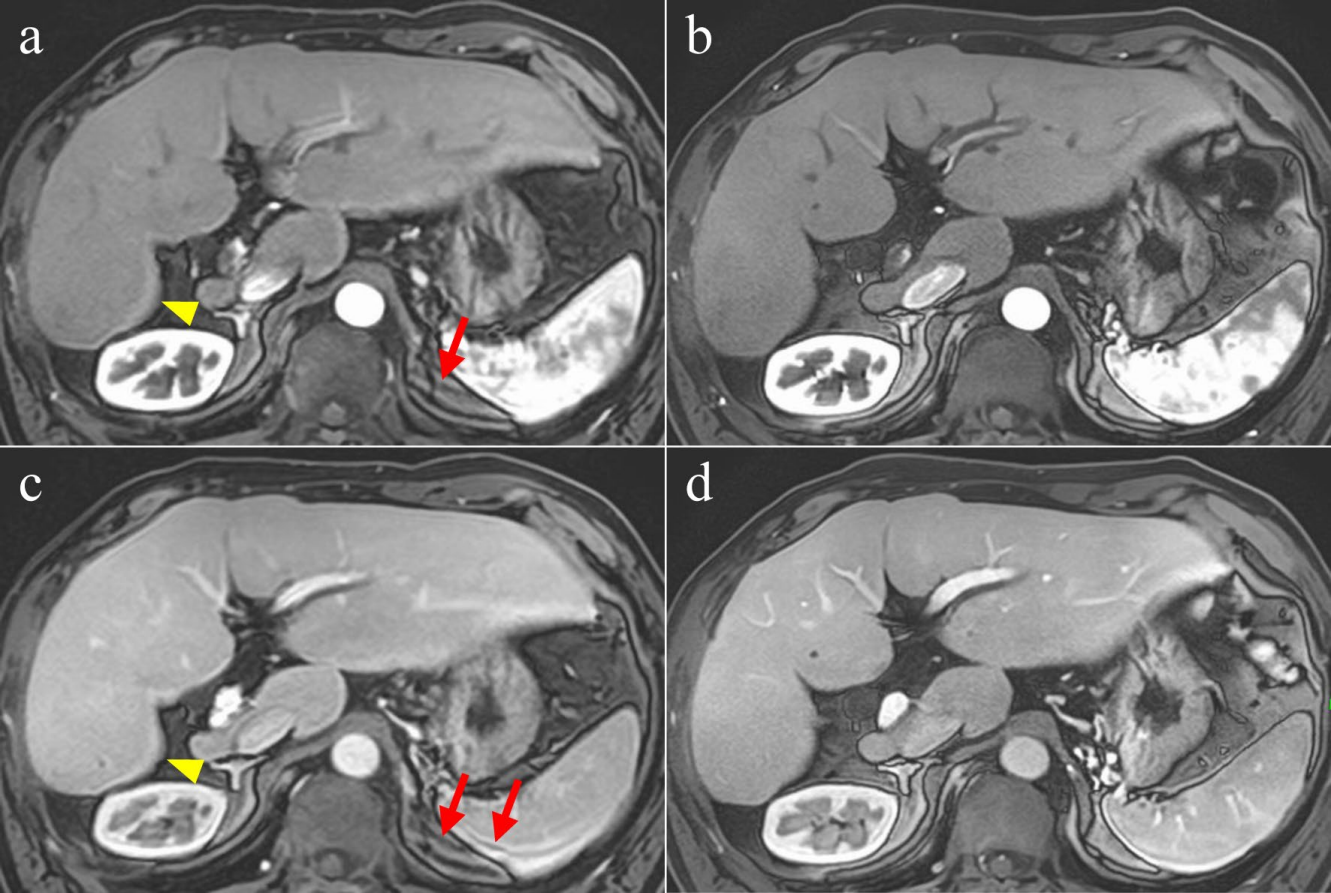
Multiphasic contrast-enhanced three-dimensional (3D) dynamic MR imaging of the abdomen during a breath holding. **a** CS with AiCE in arterial phase (AP), **b** a mFast 3D wheel using AiCE in AP. **c** CS with AiCE in portal venous phase (PVP), and **d** a mFast 3D wheel using AiCE in PVP. Visibility of intrahepatic vessels and overall image quality in the mFast 3D wheel using AiCE (**b**, **d**) were significantly better than those in CS with AiCE (**a**, **c**) in both AP and PVP images. Artifacts due to the motion (arrows) and edge enhancement (arrowheads) were observed in CS with AiCE (**a**, **c**). *CS* compressed sensing, *AiCE* advanced intelligent clear-IQ engine, *mFast* 3*D wheel* modified fast 3D mode wheel with sequential data filling

**Fig. 4:**
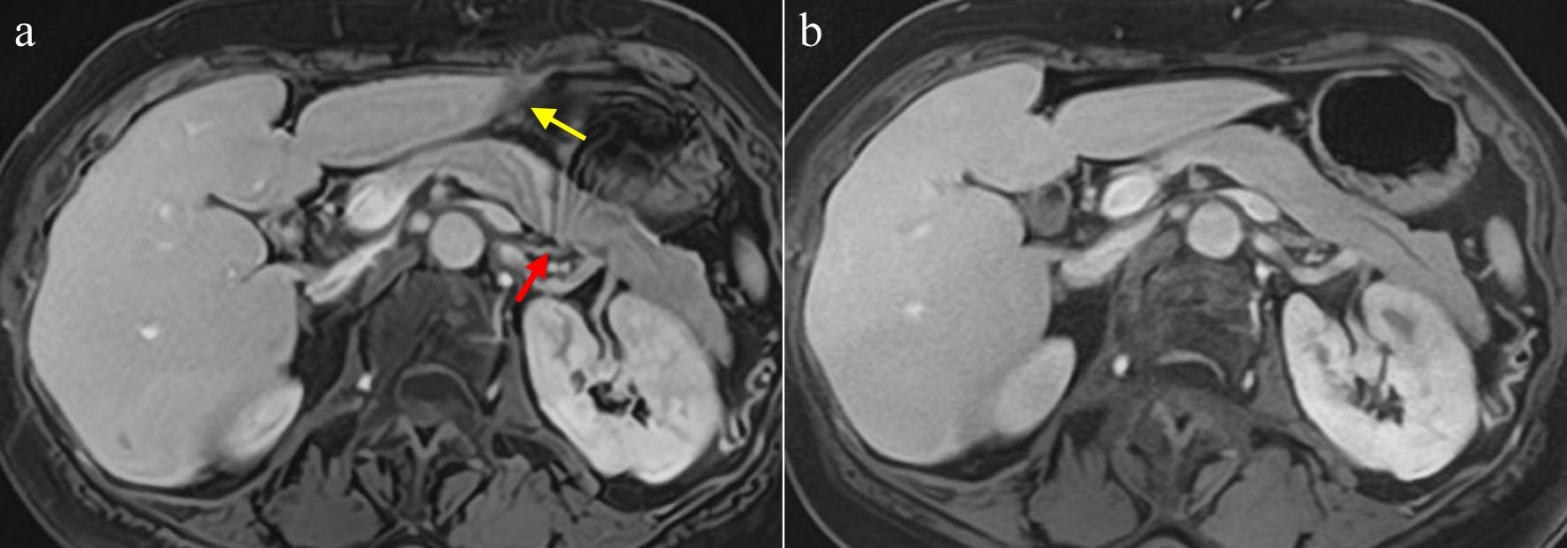
Multiphasic contrast-enhanced three-dimensional (3D) dynamic MR imaging of the abdomen during a breath holding. **a** CS with AiCE in PVP, **b** a mFast 3D wheel using AiCE in PVP. Conspicuity of abdominal organs (yellow arrow) in the mFast 3D wheel using AiCE (**b**) was better than those in CS with AiCE (**a**). Motion artifacts from the stomach (red arrow) were observed on the pancreas in CS with AiCE (**a**). *CS* compressed sensing, *AiCE* advanced intelligent clear-IQ engine, *mFast* 3*D wheel* modified fast 3D mode wheel with sequential data filling

In the qualitative analysis, a Fleiss kappa test showed moderate to substantial inter-observer agreement (motion artifact: 0.450, edge enhancement artifact: 0.525, noise: 0.610, conspicuity: 0.654, overall image quality: 0.727). The results of the visual evaluation scores of the qualitative analysis are presented in Table 3. The conspicuity of abdominal organs, intrahepatic structures, and overall image quality of the mFast 3D wheel using AiCE were significantly better in comparison to CS with AiCE (median [IQR]: 5 [0] vs. 4 [0], *p* < 0.001, and 5 [1] vs. 4 [0], *p* < 0.001, respectively) (Fig. 3). Artifacts due to motion and edge enhancement in the mFast 3D wheel using AiCE were significantly reduced in comparison to CS with AiCE (median [IQR]: 5 [0] vs. 4.5 [1], *p* < 0.001, and 5 [0] vs. 4 [1], *p* < 0.001, respectively) (Fig. 4). Conversely, image noise in CS with AiCE was significantly better in comparison to the mFast 3D wheel using AiCE (median [IQR]: 5 [1] vs. 4 [1], *p* = 0.005). There was no significant difference in the timing of the arterial phase between CS with AiCE and the mFast 3D wheel using AiCE (CS: early = 2, optimal = 38, late = 2; mFast 3D wheel: early = 1, optimal = 38, late = 3, *p* = 1.000).

**Table 3:** Comparison of visual evaluation scores in qualitative analysis

	CS	mFast 3D wheel	*p* value	Fleiss kappa
Noise	5 (1)	4 (1)	0.005	0.610
Motion artifact	4.5 (1)	5 (0)	< 0.001	0.450
Edge enhancement artifact	4 (1)	5 (0)	< 0.001	0.525
Conspicuity	4 (0)	5 (0)	< 0.001	0.654
Overall image quality	4 (0)	5 (1)	< 0.001	0.727

Data are medians, with interquartile ranges in parentheses

*CS* compressed sensing, *mFast 3D wheel* modified Fast 3D mode wheel with sequential data filling

The quantitative measurements are compared between CS with AiCE and the mFast 3D wheel using AiCE in Tables 4 and 5. There were no significant differences in the SIR of the liver and pancreas in AP or PVP images between CS with AiCE and mFast 3D wheel using AiCE. The SNR of the liver in CS with AiCE was significantly better in comparison to the mFast 3D wheel using AiCE in both AP (23.7 ± 5.5 vs 19.7 ± 4.1, *p* < 0.01) and PVP (28.7 ± 6.6 vs 25.4 ± 5.9, *p* < 0.01) images. Conversely, the SNR of the pancreas in PVP was significantly better in the mFast 3D wheel using AiCE in comparison to CS with AiCE (10.9 ± 3.8 vs 9.6 ± 3.3, *p* = 0.03). Regarding the assessment of contrast enhancement effects of the organs, there were no significant differences in the CR of the liver and pancreas in both AP and PVP images between CS with AiCE and the mFast 3D wheel using AiCE. Additionally, no significant differences were observed in the CER of the liver and pancreas on AP or PVP images between CS with AiCE and the mFast 3D wheel using AiCE. The CNR of the liver in CS with AiCE was significantly better in comparison to the mFast 3D wheel using AiCE in both AP (8.5 ± 4.7 vs 7.2 ± 3.4, *p* = 0.01) and PVP (13.1 ± 5.3 vs 11.5 ± 4.0, *p* < 0.01) images.

**Table 4:** Comparison of SIR and SNR in quantitative analysis

	SIR			SNR		
	CS	mFast 3D wheel	*p* value	CS	mFast 3D wheel	*p* value
AP						
Liver	1.62 ± 0.57	1.63 ± 0.45	0.81	23.7 ± 5.5	19.7 ± 4.1	< 0.01
Pancreas	2.33 ± 0.71	2.27 ± 0.51	0.19	9.3 ± 3.5	9.9 ± 3.4	0.19
PVP						
Liver	1.92 ± 0.58	1.89 ± 0.44	0.39	28.7 ± 6.6	25.4 ± 5.9	< 0.01
Pancreas	1.86 ± 0.60	1.79 ± 0.38	0.11	9.6 ± 3.3	10.9 ± 3.8	0.03

Data are means ± standard deviations

*SIR* signal intensity ratio, *SNR* signal-to-noise ratio, *CS* compressed sensing, *mFast* 3*D wheel* modified fast 3D mode wheel with sequential data filling, *AP* arterial phase, *PVP* portal venous phase

**Table 5:** Comparison of CR and CER in quantitative analysis

	CR				CER				CNR		
	CS	mFast 3D wheel	*p* value		CS	mFast 3D wheel	*p* value		CS	mFast 3D wheel	*p* value
AP											
Liver	0.34 ± 0.15	0.35 ± 0.13	0.81		0.20 ± 0.19	0.23 ± 0.20	0.43		8.5 ± 4.7	7.2 ± 3.4	0.01
Pancreas	0.53 ± 0.14	0.54 ± 0.11	0.17		0.66 ± 0.26	0.67 ± 0.28	0.74		5.0 ± 2.3	5.4 ± 2.1	0.50
PVP											
Liver	0.44 ± 0.14	0.45 ± 0.10	0.43		0.54 ± 0.22	0.54 ± 0.19	0.87		13.1 ± 5.3	11.5 ± 4.0	< 0.01
Pancreas	0.42 ± 0.18	0.42 ± 0.12	0.13		0.42 ± 0.16	0.42 ± 0.19	0.19		4.1 ± 2.1	4.6 ± 1.9	0.10

Data are means ± standard deviations

*CR* contrast ratio, *CER* contrast enhancement ratio, *CNR* contrast-to-noise ratio, *CS* compressed sensing, *mFast* 3*D wheel* modified fast 3D mode wheel with sequential data filling, *AP* arterial phase, *PVP* portal venous phase

## Discussion

In the qualitative analysis of this study, the mFast 3D wheel using AiCE were inferior to CS with AiCE in terms of image noise and superior in terms of artifacts (motion and edge enhancement) and conspicuity of abdominal organs and intrahepatic structures. The overall image quality in the mFast 3D wheel using AiCE was also better than in CS with AiCE, probably because observers appreciated the improvement in conspicuity and image sharpness more than image noise and contrast. In both sequences, the use of AiCE reduces image noise and improves image contrast; however, CS also incorporates a noise reduction process in its reconstruction algorithm [16, 17], which further improves the SNR and image contrast. However, in this denoising process in CS, signal loss due to threshold settings during wavelet transformation occurs, causing image blurring, which degrades conspicuity and image sharpness [17–20]. Conversely, the mFast 3D wheel using AiCE were reconstructed with in-plane high-spatial resolution, and data were collected in a wheel shape, thinning out the four corners of the k-space to reduce acquisition time [10, 21]. Additionally, the data is filled in the sequential direction of the k-space for both slice encoding and phase encoding during wheel-shaped data sampling. In this sequential data filling, a stable signal after a steady-state transition is laid in the center of the k-space, resulting in reduced blurring and the improvement of conspicuity, sharpness, and image quality. The edge enhancement artifacts observed in CS with AiCE are likely chemical shift-related artifacts. During random sampling in CS, data are collected in the order of phase-encoding direction to slice-encode direction. In this case, a chemical shift from the slice direction tends to appear in the imaging plane, resulting in the appearance of edge enhancement artifacts at organ margins.

Quantitatively, the SNR and CNR of the liver was significantly better in CS with AiCE than in the mFast 3D wheel using AiCE. This can be explained by the more effective denoising effect of the combination of CS and AiCE. In contrast, the SNR of the pancreas in AP images did not differ to a statistically significant extent different between CS with AiCE and the mFast 3D wheel using AiCE. The pancreas is a hypervascular organ, and it was shown that sufficient SNR is maintained in the mFast 3D wheel using AiCE when increased contrast enhancement can be expected in the AP, suggesting that it can be applied to the detection and diagnosis of hypovascular panceatic lesions. The SNR of the pancreas in PVP was rather significantly better in the mFast 3D wheel using AiCE than in CS with AiCE. The decreased SNR in CS may be due to increased SD values resulting from unavoidable motion artifacts in ROI measurements because of its nature as a small organ.

The SIR, CR, and CER of the liver and pancreas in the mFast 3D wheel using AiCE were comparable to those in CS with AiCE in both AP and PVP images. In CE dynamic MR of the abdomen, the ability to obtain an adequate contrast enhancement effect in the organs is of utmost clinical importance [22, 23]. In this respect, the mFast 3D wheel using AiCE is a clinically applicable imaging method that can improve image conspicuity, sharpness, and overall image quality, in addition to providing sufficient contrast enhancement effects.

The present study was associated with several limitations. First, a selection bias may be unavoidable due to the retrospective study design and small number of patients. Second, the AiCE level was fixed at 4 for both sequences. Therefore, further studies with different denoising levels in AiCE are required. Finally, no comparison or evaluation of the ability to detect or differentiate between solid lesions was performed. The reason for this is that the study compared CE dynamic MR images taken on different dates within 6 months in the same patient. Had solid lesions been included, the vascularity or nature of the nodules may have changed within the study period, which would not have allowed an accurate comparison. Therefore, we focused on the intra-individual comparison of the image quality and contrast enhancement effects of the abdominal organs. Further studies in larger populations with solid lesions are required to validate our results.

In conclusion, the mFast 3D wheel using AiCE as a deep learning denoising technique improved the conspicuity of abdominal organs and intrahepatic structures and overall image quality with sufficient contrast enhancement effects. Therefore, its application will be clinically feasible for high spatial- and contrast-resolution BH 3D CE dynamic MR of the abdomen.

## Abbreviations

AiCE: Advanced intelligent clear-IQ engine
BH: Breath-hold
CE: Contrast-enhanced
CER: Contrast enhancement ratio
CNR: Contrast-to-noise ratio
CR: Contrast ratio
CS: Compressed sensing
mFast 3D wheel: Modified Fast 3D mode wheel with sequential data filling
SIR: Signal intensity ratio
SNR: Signal-to-noise ratio
